# IDPpi: Protein-Protein Interaction Analyses of Human Intrinsically Disordered Proteins

**DOI:** 10.1038/s41598-018-28815-x

**Published:** 2018-07-12

**Authors:** Vladimir Perovic, Neven Sumonja, Lindsey A. Marsh, Sandro Radovanovic, Milan Vukicevic, Stefan G. E. Roberts, Nevena Veljkovic

**Affiliations:** 1 Centre for Multidisciplinary Research and Engineering, Vinca Institute of Nuclear Sciences, University of Belgrade, Belgrade, Serbia; 2 School of Cellular and Molecular Medicine, University of Bristol, Bristol, UK; 3 Centre for business decision making, Faculty of organizational Sciences, University of Belgrade, Belgrade, Serbia

## Abstract

Intrinsically disordered proteins (IDPs) are characterized by the lack of a fixed tertiary structure and are involved in the regulation of key biological processes via binding to multiple protein partners. IDPs are malleable, adapting to structurally different partners, and this flexibility stems from features encoded in the primary structure. The assumption that universal sequence information will facilitate coverage of the sparse zones of the human interactome motivated us to explore the possibility of predicting protein-protein interactions (PPIs) that involve IDPs based on sequence characteristics. We developed a method that relies on features of the interacting and non-interacting protein pairs and utilizes machine learning to classify and predict IDP PPIs. Consideration of both sequence determinants specific for conformational organizations and the multiplicity of IDP interactions in the training phase ensured a reliable approach that is superior to current state-of-the-art methods. By applying a strict evaluation procedure, we confirm that our method predicts interactions of the IDP of interest even on the proteome-scale. This service is provided as a web tool to expedite the discovery of new interactions and IDP functions with enhanced efficiency.

## Introduction

Protein–protein interactions (PPIs) underlie many processes essential to living organisms and hence, are fundamental to the functional annotation of proteins. So far, we have been acquiring genomic information much faster than knowledge and understanding of protein interactions. Low-throughput experimental studies that reveal PPIs are not only expensive and labour-intensive, but also suffer from bias in favour of well-studied proteins. Under the circumstances, *in silico* prediction methods are a valuable complement that can prioritize candidates for rigorous experimental testing or eliminate those less likely to interact. To this end, computational approaches that utilize machine learning classifiers and rely on available PPI data predict uncovered segments of the interactome at an accuracy similar to those of high-throughput techniques and appear to be particularly useful for their comprehensiveness^1–3^. These methods operate on protein sequences converted into feature vectors, so they are universal and easily applicable, which is an advantage over computationally-intensive structure-based molecular docking methods. However, supervised machine learning algorithms for PPI predictions suffer from inherent limitations in foreseeing interactions of components that were unseen by the model during the training process. Several papers have reflected on sustainable estimates of PPI prediction performances and their ability to generalize to physiological environments^4,5^.

Intrinsically disordered proteins (IDPs) represent a structural class of proteins that do not have well-defined tertiary structures in several regions or throughout the entire sequence^6–8^. This conformational plasticity is associated with sequence compositional characteristics including low proportions of bulky hydrophobic amino acids and a high content of charged and hydrophilic residues^9^. The ability of a single IDP to bind to several structurally dissimilar partners, complemented with the ability of many different IDPs to bind to a single partner, results in exceptional binding promiscuity^10^. Coupled folding and binding, one inherent property of IDPs, is associated with their propensity to form low-affinity complexes which govern or maintain signalling processes^11^. Thus, many IDPs act as hubs in interaction networks and have central roles in the regulation of signalling pathways^12^. On the other hand, in fuzzy complexes, bound IDPs might remain fully or partially disordered^13^.

IDPs habitually have multiple functions and underpin key cellular processes, including the regulation of transcription, translation and the cell cycle. In addition to regulatory roles, IDPs are key players in many other biological processes such as molecular recognition, binding of small molecules, and the organization of chromatin^14–17^. IDPs are the prevailing protein class associated with noncommunicable diseases^18,19^ and therefore, mapping the interactome of IDPs will lead to improved understanding of disease mechanisms and provide the platform for novel therapeutic approaches^20,21^. Thus, it is an important task of computational biology to provide model PPI networks of IDPs and to enable reliable predictions of candidate interactors. The flexible structure of IDPs imposes restrictions on the application of precise computational methods for modelling interactions based on docking. This is because they rely on putative binding modes according to favourable interaction energies and surface complementarities, thus requiring candidates with stable, well-defined, globular three-dimensional structures^22,23^. On the other hand, data-driven methods have no inherent barriers to respond to this task, but require methods to prevent bias in the training while carrying out a realistic estimation of performance.

In this study, we used compositional features to decode amino acid strings and implemented machine learning algorithms to predict binary interactions that involve IDPs. With a strict evaluation procedure and attention to potential setbacks intrinsic to data-based methods, we demonstrate that our classifier grasps key characteristics of PPIs and is capable of predicting interactions of an IDP of interest with significant efficiency.

## Results and Discussion

### Overview of the data sets

**Figure 1:**
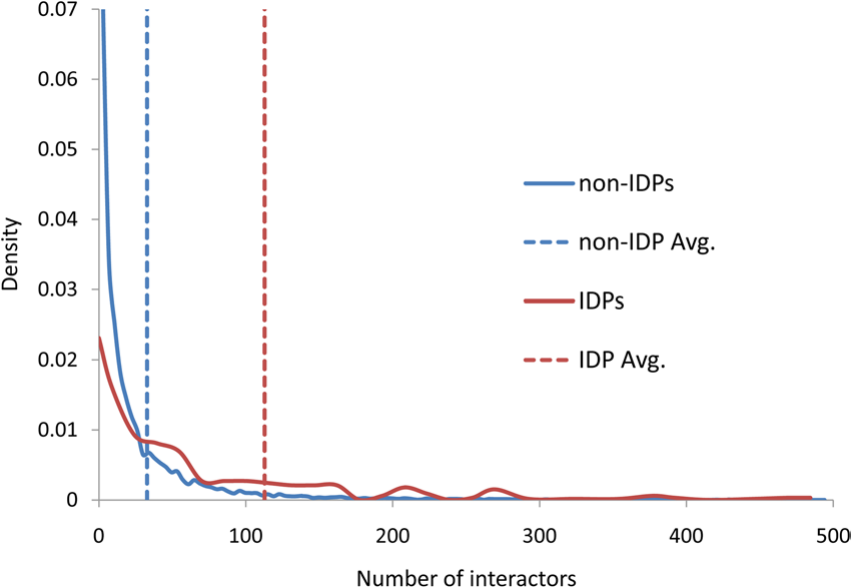
Number of interactors - density curves for the interactions in the HIPPIE database. IDPs density curve is represented by a red line and ordered proteins are denoted non-IDPs and their density curve is a blue line. The average number of interactions per IDP is 112.77; The average number of interactions per ordered protein is 31.58.

The database of protein disorder (DisProt) collects data on intrinsically disordered proteins and its current version holds information on 237 human entities^24^. Some of these proteins are entirely disordered, whereas others contain both disordered regions and globular domains. In this study, we utilized this dataset as a representative of human disordered proteins and the term IDP is used as a universal expression to denote an intrinsically disordered human protein. The interactome made of binary PPIs that involve at least one IDP from the DisProt was extracted from the Human Integrated Protein-Protein Interaction rEference (HIPPIE) database^25^, a source specialized in human interactions, which combines information on PPIs with experimental annotation from ten primary repositories. The HIPPIE data are manually curated and associated with a quality scoring scheme that we used to eliminate less reliable data. As mentioned earlier, the complexity of the disorder-based interactomes is increased through the capacity of a single IDP to bind to multiple partners. Several studies have shown a correlation between the disorder of a protein and the number of its partners^26–28^. Indeed, according to the statistics of the version of HIPPIE that we used, the number of interactions per IDP is approximately 3.5 times higher than that of PPIs per ordered protein. Specifically, the average number of interactions per IDP is 112.77, whereas the average number of interactions per ordered protein is 31.58 (Fig. 1).

In such an environment, the concern that hub proteins will dominate the PPI prediction algorithm is realistic and reasonable. In order to preclude representational bias in the learning process, we performed a balanced sampling of negative PPIs. Thus, all sequences, components of protein pairs appeared evenly in the positive and negative parts of the training set. A common approach towards building a negative dataset is to sample protein pairs that are not known to interact. The number of false negatives in such collections is considered low because number of protein pairs significantly exceeds the number of recognized interactions. For the test sets, negatives were subsampled randomly as required for evaluation of the PPI prediction method at a population-level^29,30^.

**Figure 2:**
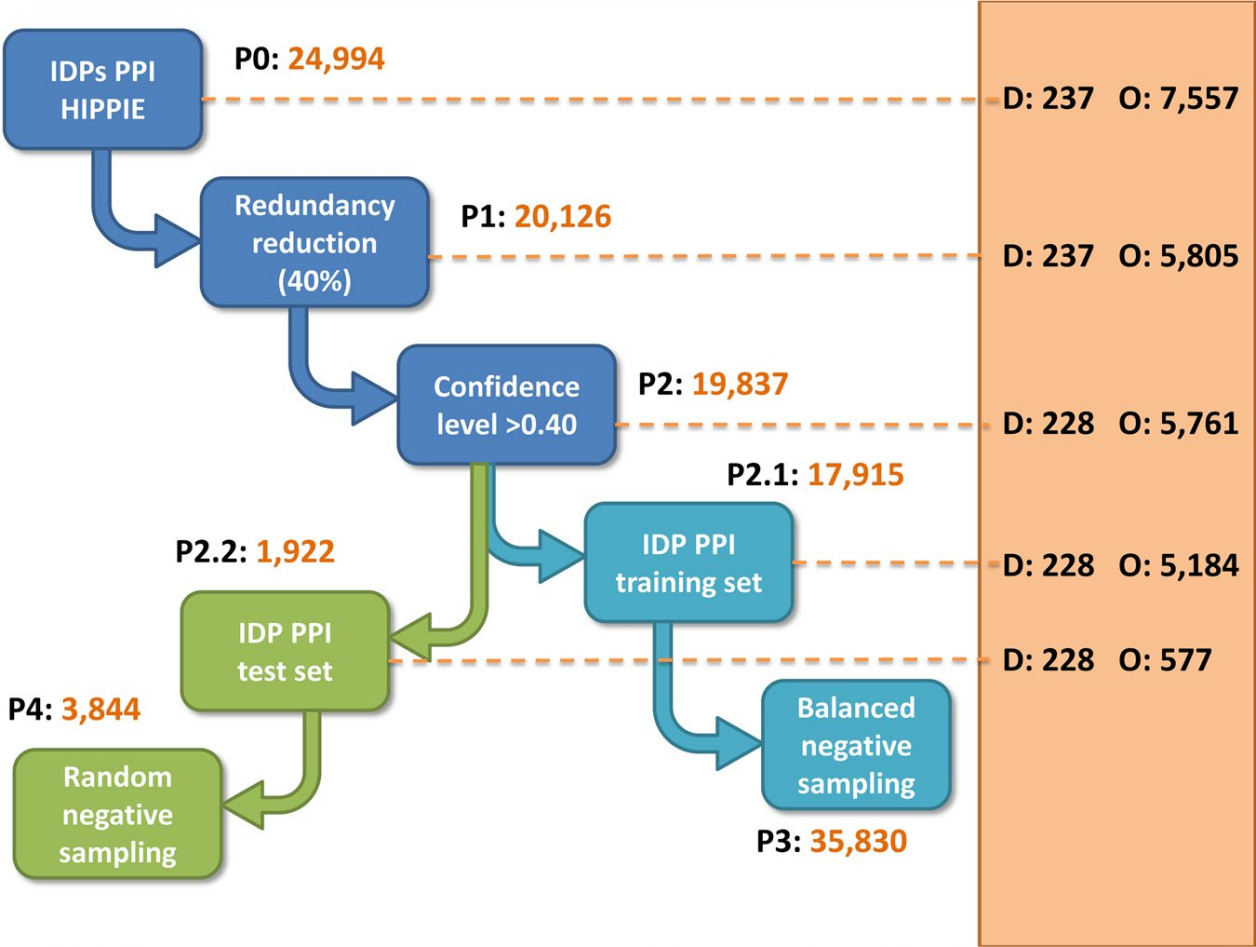
Flowchart to show the process of building data sets. Squares describe the pre-processing steps executed to obtain datasets for training (blue) and testing (green). Left block (white) displays the number of elements of PPI datasets, denoted P-sets. Right block (orange) displays the numbers of protein components to build protein pairs, IDPs (D-sets) and ordered proteins (O-sets).

As a first step in generating datasets we retrieved 24,994 interactions with at least one IDP included (Fig. 2). Of note, the number of interactions per IDP in our dataset is close to the average per disordered protein in the human proteome. The redundancy among the initial set of 7,557 interactors was reduced by removing sequences with identity levels higher than 40% by clustering analysis using the CD-HIT^31^. The remaining dataset encompassed 20,216 interactions involving 5,805 unique ordered sequences. Next, we removed interactions that were less reliable in experimental annotation. The remaining items are partitioned randomly into training and test sets for holdout validation. We repeated this 5 times ensuring that the test pairs share only one IDP component with the training set. The entire list of 5 training sets can be found as Supplementary Tables S1–5.

It is estimated that at least 30% of the human proteome contains disordered regions and consequently the IDP dataset used in our study covers a tiny part of the human disordered proteome. Nevertheless, according to the number of known interactions it seems that the dataset used here contains well-explored IDPs that are functionally important. We anticipate that future upgrades of the DisProt and HIPPIE databases will significantly contribute to information on human IDPs and that these prospective improvements to datasets used for testing and training will positively influence the quality of the predictions.

### Human Disorder PPI predictions

IDPs exhibit highly specific sequence composition^32^ across both the overall structure^33^ and binding domains^34^. In PPIs that involve IDPs the binding energy depends on residue composition, interface sizes, and flexibility of the interaction partners^35^. This motivated us to explore the use of residue composition in engineering features associated with the selection of binding partners. Two types of amino acid composition were employed in depicting full-length sequences. Firstly, the alphabetic series was transformed into feature vectors by means of the dipeptide composition which views every two consecutive amino acids as a single element and counts the frequency of all of dipeptide configurations. Second, we used pseudo amino acid composition (PAAC) and incorporated five amino acid propensity scales: the TOP-IDP scale which ranks residues by the their propensity to endorse order or disorder^36^, B-values which quantitates the flexibility parameters for each residue surrounded by two inflexible neighbours^37^, the FoldUnfold scale which represents the capacity of amino acid residues to form a sufficient number of contacts in a globular state^38^, the DisProt scale which is based on the statistical difference in the residue composition of ordered proteins and IDPs as in^36^ and the net charge scale^39^. The PAAC composition contains both a conventional amino acid composition and factors that incorporate sequence-order information via feature correlation functions^40^. Protein pairs were vectorized by a single sequence vectors concatenation and represented as 940-dimensional vectors.

In a previous study Park and Marcotte^4^ explored the consequences of the component-level overlap between the training and the test sets by distinguishing three types of test-pairs. They did this according to whether they share both, one or no sequence/s with the protein pairs in the training set. This approach demonstrated that each method achieved the best performances for the test sets that shared both components with the training and the worst for those that shared none. In the present study, which aimed to assess how our method executes on proteome-scale predictions, we performed evaluation experiments on test pairs which shared only the IDP component with a training dataset. Firstly, we used 10-fold cross-validation to select the best performing machine learning algorithm among Random Forests (RF), Gradient Boosting Machine (GBM), Support Vector Machine (SVM), and Generalized Linear Model (GLM). According to averaged results on 10 test folds (Supplementary Table S6) the best performing model utilizes the RF algorithm. We denoted this model IDPpi and retrained it on IDP datasets (Supplementary Tables S7–S11) with optimized RF for comparative evaluation on hold-out test sets. In the test sets one component in every protein pair was an IDP, whereas another component was randomly selected from sequences that were completely new to the model and bore less than 40% similarity to those used for training. In this way we estimated IDPpi performance on forecasting interactions even with proteins yet to be discovered. The predictive performance of IDPpi is compared with state-of the-art approaches M1^41^, M2^42^ and M3^43^ that were previously used in the aforementioned study^4^. Comparative testing was repeated 5 times on independent test sets (Supplementary Tables S12–S16) and averaged results confirmed that IDPpi outperforms all other methods (Table 1).

**Table 1:** Comparison of the prediction performances between our proposed method, IDPpi and other state-of-the-art methods: M1^41^, M2^42^ and M3^43^.

Method	AUC	AUPRC	ACC	F	MCC
IDPpi	0.746 ± 0.017	0.734 ± 0.020	0.670 ± 0.015	0.633 ± 0.021	0.348 ± 0.028
M1	0.688 ± 0.017	0.697 ± 0.018	0.638 ± 0.013	0.590 ± 0.022	0.285 ± 0.025
M2	0.637 ± 0.014	0.613 ± 0.012	0.593 ± 0.010	0.553 ± 0.019	0.190 ± 0.021
M3	0.627 ± 0.011	0.643 ± 0.014	0.599 ± 0.008	0.518 ± 0.013	0.211 ± 0.017

The next step was to assess how the model performs in a real-life environment where the number of negatives significantly surpasses the number of positives. To this end, we tested the performance of the model on test sets (Supplementary Tables S22, S23) in which the number of positives (N) was accompanied by the number of negatives augmented several times and yet, the IDPpi model outperforms other methods. As shown in Table 2, AUCs and ACCs remained almost the same between the 10 N and the 100 N sets, whereas AUPRC, which is significantly more sensitive to absolute numbers of false positives, considerably decreased.

**Table 2:** Evaluation using a negative subsets randomly chosen from the negative set, where N is the size of the positive set.

	10 N			100 N		
	AUC	AUPRC	ACC	AUC	AUPRC	ACC
IDP-PPI	0.745	0.237	0.740	0.748	0.050	0.757
M1	0.691	0.217	0.724	0.692	0.048	0.737
M2	0.645	0.140	0.648	0.646	0.025	0.657
M3	0.624	0.163	0.740	0.624	0.032	0.763

**Figure 3:**
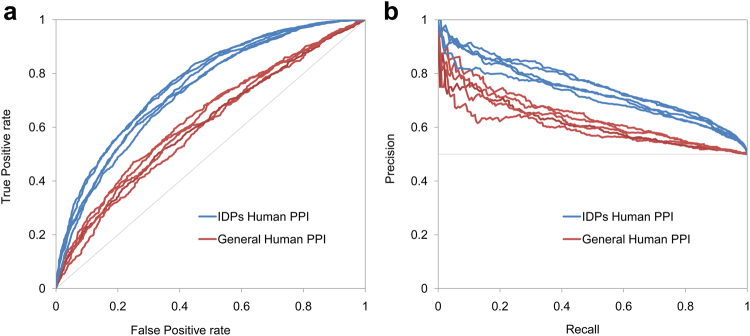
Comparison of predictive performances through (**a**) ROC curves and (**b**) precision/recall plots. Performances are evaluated across 5 IDP test sets (blue) and 5 general human PPI test sets (red). In every test set the protein pairs shared exactly one component with the training dataset. In case of IDP test sets these components were IDPs.

Mészáros and colleagues have reported previously that the interaction surfaces of IDPs represent a distinct employment of the principles of protein–protein recognition^34^. Here, we sought to demonstrate that our model is fine-tuned to predict interactions that involve IDPs. To this end, 5 human PPI datasets^4^ (Supplementary Tables S1–S5) were used for training of the IDPpi and test sets (Supplementary Tables S17–S21) in which the protein pairs shared only one component with the training datasets. We compared the model performances (Fig. 3) and confirmed its enhanced performance in predicting interactions with IDP components. As a corollary, our model offers significant improvements to the accuracy and coverage of human IDP interactions compared to general proteome-wide models. It may serve as a helpful resource for understanding the core cellular functions and mechanistic foundations of human diseases and therefore, we offer it to the public as a convenient website: http://www.vin.bg.ac.rs/180/tools/dispred which can test potential interactions of IDPs that are expertly curated and belong to the human corpus of the DisProt database. The user can select an IDP of interest from the dropdown menu in the left combo-box, and in the right window the user is allowed to input up to 100 prospective interactors. There are no restrictions in selecting potential partners irrespective of their structural characteristics, evidence level or species of origin. Hence, their sequences are required to be input manually in FASTA format. In the query webpage we integrated cross-links to UniProt and DisProt entries that correspond to the selected intrinsically disordered protein. Additionally, the cross-link is provided on the NextProt dedicated page that displays information on the regions, variants and binding domains of the query protein. NextProt is a highly reliable manually curated knowledge platform that ensures up-to-date information on human proteins^44^.

We are aware that by utilizing the reference isoforms, and ignoring the effects of alternative splicing and post-translational modifications, protein interactome mappings fail to consider the entire human proteome complexity, particularly of the IDP interactome sub-element. Even so, solving the problem of large-scale predictions of this IDP subset paves the way to more complex and comprehensive sequence-based PPI predictions for this protein class.

### Illustrative example: Interactome Map of BASP1

**Figure 4:**
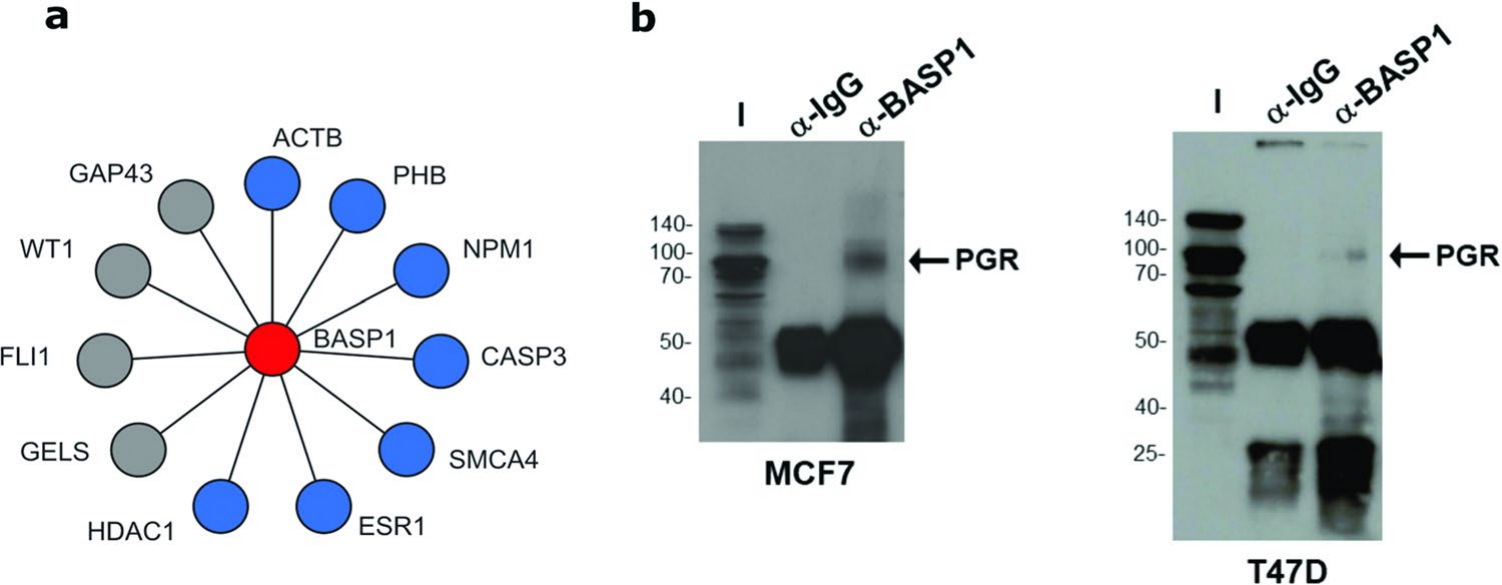
(**a**) Case study for true positive predictions. Confirmed BASP1 interactions: HDAC1, ACTB, PHB, CASP3, SMCA4, ESR1, and NPM1 (blue nods) are predicted with IDPpi, whereas WT1, GELS, FLI1 and GAP43 (grey nods) are predicted negative by the IDPpi. (**b**) Predicted interaction between BASP1 and progesterone receptor, PRGR: *In vivo* binding confirmation. Nuclear extracts were prepared from either MCF7 or T47D breast cancer cells. The nuclear extracts were then subjected to immunoprecipitation with either control antibodies (a-IgG) or BASP1 antibodies (a-BASP1). The immunoprecipitates were subjected to SDS-PAGE alongside 5% of input nuclear extract (I) and immunoblotted with anti-PGR antibodies. Molecular weight markers (kDa) are shown on the left.

Transcription and transcriptional regulation are among the most notable molecular functions that require proteins with flexible structures to underpin precisely timed and context specific processes. The brain acid-soluble protein-1 (BASP1) is a transcriptional cofactor^45^ characterized as an intrinsically disordered structure throughout its entire sequence^46^. BASP1 is widely expressed in embryonic and adult tissues and the BASP1 gene is silenced in several tumour types^47–49^. The roles of BASP1 in health and disease are not yet well understood and we sought here to use IDPpi to predict previously unknown interactors and to connect this information with functional insights. Firstly, we examined the ability of our model to predict already known BASP1 interactors from the literature that were not used in the learning process. IDPpi accurately predicted 7 out of 11 known positives (Supplementary Table S24) among which are some of the highest-scoring IDPpi candidates (Fig. 4a): Histone Deacetylase 1 (HDAC1)^50^, Actin Beta (ACTB)^51^, Nucleophosmin 1 (NPM)^51^ and caspase 3 (CASP3)^52^. We further experimentally evaluated and confirmed one interesting novel candidate interaction between BASP1 and the progesterone receptor, PRGR (Fig. 4b). Previous studies have shown that BASP1 acts as a transcription cofactor for WT1^53^, v-myc^54^ and estrogen receptor (ESR1)^51^. Our finding that BASP1 associates with PRGR and that interactions are predicted with several other DNA-binding transcription factors suggests that BASP1 is likely to be a widely deployed transcription cofactor. Previous studies have reported that BASP1 binds to chromatin remodelling factors (HDAC1, SMCA4)^50^ suggesting that BASP1 acts as an interface between the DNA-bound transcription factors and proteins that modulate the transcription process. Indeed, the analysis presented here found that BASP1 potentially also interacts with several chromatin remodelling factors.

**Figure 5:**
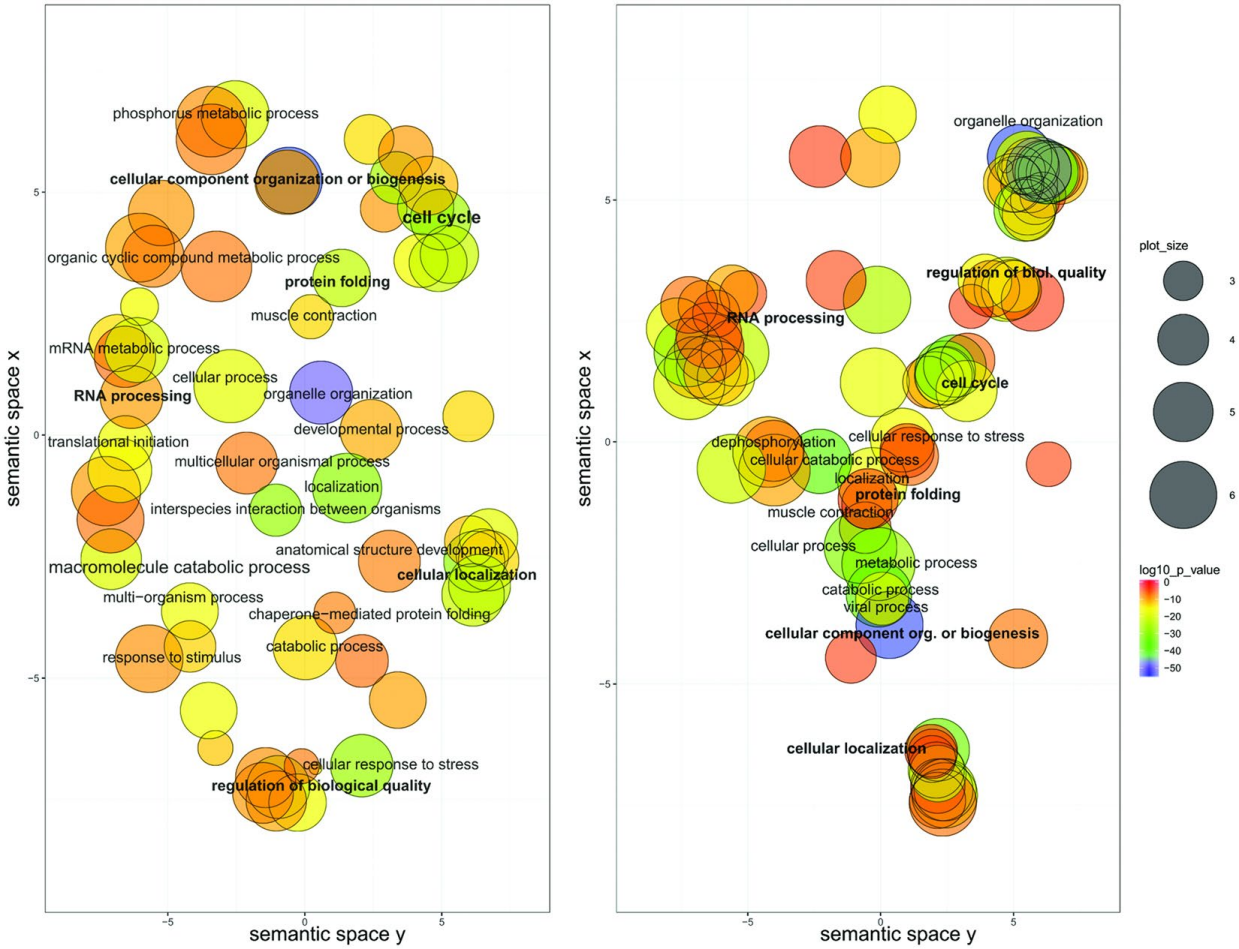
Enrichment analysis of GO terms related to biological processes in a set of BASP1 established (left) and predicted (right) interactions. Significantly enriched GO terms are plotted in the semantic space so that similar terms are represented close to one another. Markers are scaled and colored according to the log10 of the p-value for the significance of each term. Blue circles are highly significant, while red circles are less so.

In order to understand the global functional perspective that is suggested by the projected BASP1 interactome, we analysed the enrichment of GO terms defined with interactors predicted by IDPpi. Further, we compared it to the list of enriched terms in a set of known positives and singled out correctly predicted functions and prospective BASP1 engagements. Analysed terms belong to the sub-ontology of biological processes (BP). Enrichment of two sets (Supplementary Tables S25, S26) was determined by BINGO^55^ and the terms with scores above the predefined cut-off are presented by the visualisation tool, Revigo^56^. In these scatter plots (Fig. 5), GO term nodes that are plotted nearer to one another are more similar in semantic space, whereas node colours showed a degree of enrichment. According to our findings, the predicted interactions determine that GO terms point toward processes such as cellular component organisation, RNA metabolic processes and the cell cycle (Fig. 5, right panel). This aligns with terms that come out of the list of known interactors (Fig. 5, left panel). The predicted nodes that are grouped in the semantic space point toward specific functions that are yet to be explored for BASP1, such as viral processes or protein folding (Fig. 5, right panel), both known to depend on proteins with flexible structures^12^. Note that the term “metabolic processes” that is otherwise under-represented among IDP-related categories of GO^57^, also emerged.

**Figure 6:**
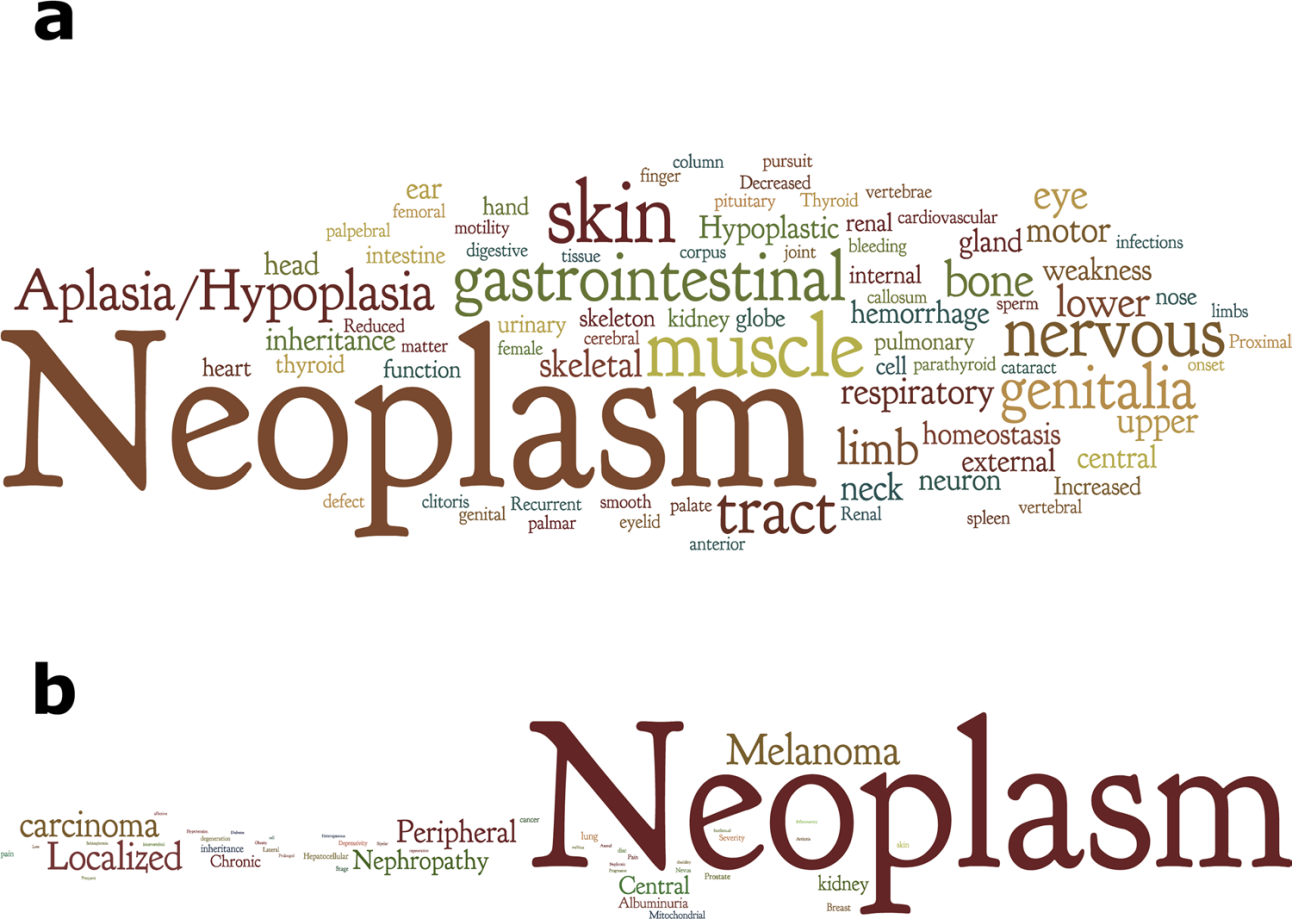
Tag-clouds representing disease terms (**a**) over-represented among 1000 top-scoring IDPI predicted interactors and (**b**) co-mentioned with BASP1 in the literature. The tag-cloud displays more frequently appearing terms using larger font sizes. Neoplasm is the dominant term in both presentations, whereas melanoma and skin are related terms.

Finally, we used a list of predicted BASP1 interactors to point towards diseases in which BASP1 might play a role. To this end, Human Phenotype Ontology (HPO) which links human genes with diseases and phenotypes^58^ assisted in revealing clinical abnormalities associated with genes of interest. We observed annotations for 274 among the 1000 highest-scoring candidate BASP1 interactors in the HPO largest sub-ontology, phenotypic abnormality and drew a tag-cloud to emphasize over-represented concepts (Fig. 6a). According to the presented results, words that are most frequent, apart from neoplasm, indicate organs and tissues of diseases such as skin, muscle, nervous system, gastrointestinal tract and genitalia (Supplementary Table S27). We compared these findings with the corpus of HPO terms associated with BASP1 in the literature, thus far (Fig. 6b). For each PubMed article that contained the word “BASP1” in the title and/or in abstract, we used the NCBO Annotator Web service^59^ to identify mentions of disease terms from the HPO in the text. Over-represented terms linked to BASP1 found by the Annotator (Supplementary Table S28) are displayed in Fig. 6b. One noticeable match among predicted and literature terms are skin cancer and melanoma. BASP1 has been reported to play a role in melanoma pathogenesis because its expression is suppressed in melanoma^60^, while Kaehler and colleagues demonstrated an association between increased BASP1 expression and improved melanoma survival^61^. Another correctly implied association connects mitochondrial disease in the literature tag-cloud and entities that express common clinical features of mitochondrial disease in the predicted cloud such as muscle, eye and nerve^62^.

Thus, we anticipate that other over-represented terms in Fig. 6a may point towards novel disease conditions connected with BASP1 that remain to be explored.

## Concluding Remarks

IDPs are distinct due to their compositional bias which influences their binding propensities and selection of partners. This motivated us to develop IDPpi, a method for PPI predictions which utilizes supervised machine learning algorithms and compositional content together with the distribution of features associated with the promotion of structural disorder along the sequence string. A subset of human IDPs that has been used for training is functionally important and well-explored and thus, its interactions provided a basis solid enough to develop a method fine-tuned to predict PPIs of this particular IDP dataset. The evaluation presented proves that IDPpi efficiently predicts interactions with proteins that were not used in the training process, which implies that this model can produce reliable predictions even on a proteome-scale. Further improvements of IDPpi will primarily encompass inclusion of new IDPs as prediction targets after an upgrade of the DisProt repository. In the long run, a computationally efficient and reliable approach implemented in IDPpi will serve as a solid foundation for a method that will reveal the full complexity of the IDP interactome by considering the effects of alternative splicing and PTM on protein-binding abilities. By introducing additional layers of information that will encompass a significant number of isoform- and PTM-specific interactions to the current training and testing datasets we will be able in the future to overcome limitations imposed by present data coverage. We anticipate that IDPpi may contribute not only to uncovering the human interactome but also to a significantly enhanced understanding of the diverse roles of IDPs and therefore we offer it as a simple and convenient web tool.

## Materials and Methods

### Datasets

The set of human disordered proteins and human proteins with disordered regions sequences were retrieved from the DisProt^24^ and UniProt/Swiss-Prot. The PPI dataset was obtained by searching the content of the HIPPIE v2.0^25^ for interaction partners of IDPs. Given that the HIPPIE database confers interacting partners only through gene name identifiers the isoform involved in the interaction is not known and therefore the sets were constructed from reference isoforms. As a result, the tau protein and calcyclin-binding protein that are present in DisProt only as alternative isoforms were not considered in this study. This list was further refined by the following steps: (1) proteins containing less than 50 amino acid were removed to filter out small proteins and protein fragments. The PPI dataset was obtained by searching the content of the HIPPIE v2.0^25^ for interaction partners of IDPs. The dataset of interaction partners was cleared of sequences that correspond to protein names containing the words ‘putative’, ‘potential’ or ‘uncharacterized’. The non-redundant subset of interaction partners was generated at the sequence identity level of 40% by clustering analysis using the CD-Hit^31^. Negative PPI data were formed from all human protein pairs containing exactly one IDP component. No negative PPI was listed as a positive in the full Hippie database.

### Protein interaction features

Sequences and protein pairs were represented by features derived from: (i) pseudo amino acid composition (PAAC)^40^ and (ii) dipeptide composition. PAAC combines conventional amino acid composition and sequence order correlates of various amino acid features. Amino acid scales that we utilized to encode sequences are provided in the Supplementary Table S29. In this way PAAC vectorizes a protein into a 20 + λ-feature vector, in which the first 20 components are the conventional amino acid composition and the rest depend on the maximum value correlation tier, λ. In the current study, the λ value was set to 50 which established the minimum sequence length as 51. In this way sequences were represented by 70 PAAC components.

Sequences were presented with dipeptide composition which takes every two consecutive amino acids as a single unit and counts the frequency of all of the dipeptide patterns. It then represents each sequence with a fixed length vector of 400.

Protein interactions were vectorized by concatenation of the vector representations for both proteins from the interaction pair. Therefore, the total number of dimensions of vector representation for single protein interaction is 940.

### Classification algorithms

For generating the classification model we used (i) tree based classifiers: random forests method^63^ and gradient boosting machine^64^; (ii) linear classifier, generalized linear model^65^ and (iii) kernel based support vector machine^66^. The process of modelling was carried out using RStudio integrated development environment^67^, while the software was implemented using R and JAVA programming languages. In order to determine whether the classifiers were able to learn general concepts or failed to generalize well on unseen data (overfit), we performed a ten-fold cross-validation over general protein datasets (Supplementary Tables S1–S5) provided in the study of Park and Marcotte^4^. These results were used to select the best performing machine learning algorithm. The best performing model was further retrained on IDP datasets (Supplementary Tables S7–S11) with optimized RF for further evaluation on hold-out test sets (Supplementary Tables S12–S16) that corresponded to the C2 class as described in^4^.

As a performance metrics we used the area under receiver operating characteristic curve (AUC) or recall-fallout plot, area under precision-recall plots (AUPRC), and accuracy (ACC), F score, precision, recall (sensitivity or true positive rate), fall-out (false positive rate) and Matthews correlation coefficient (MCC), calculated with the 0.5 threshold value.

Cartesian grid search and random search^68^ were used for hyperparameter optimization for each of the machine learning methods. For Random Forest following hyperparameters were tuned: number of trees, maximum tree depth, minimum number of observations for a leaf, histogram type, number of bins for the histogram, row sampling rate and number of columns to randomly select at each level; for Gradient Boosting Machine: number of trees, maximum tree depth, minimum number of observations for a leaf, histogram type, number of bins for the histogram, learning rate, row sampling rate and loss function; for Generalized Linear Model: model type, solver, penalized model, regularization penalties, beta epsilon value and maximum number of active predictors; for Support Vector Machine: C and gamma parameters and kernel function.

### State of the art methods for comparison

The method denoted M1 was developed by Martin *et al*.^41^ and relies on a signature product that is implemented within a SVM classifier as a kernel function. Next, M2, the method developed by Guo and coworkers^42^ utilizes sequences represented by a feature vector based on autocorrelation values of 7 physicochemical scales. The feature vectors are then concatenated for a protein pair and classified by an SVM. Finally, M3 method^43^ represented a protein sequence vectorized by a tri-peptide composition.

### Experimental testing

MCF7 and T47D cells were maintained as described in^51^. Immunoprecipitation was performed as described in^53^. PGR antibody was from Santa Cruz Biotechnology (Sc-538). BASP1 antibodies were described in Carpenter *et al*.^53^.

### Gene ontology and enrichment analysis

Biological process (BP) gene ontology (GO) term over-representation was calculated using BiNGO v3.0.3in Cytoscape v3.4.0, employing the hypergeometric test and applying a significance cutoff of FDR-adjusted P-value ≤ 0.05. The 17,531 human genes were used as the reference set, and the GO ontology and annotation files used were downloaded on Oct. 25, 2017. The output from BiNGO^55^ was imported into ReviGO^56^ to depict the list of significant GO terms by selecting representative subsets using a simple clustering algorithm that relies on measures of semantic similarity between terms.

### Annotating text using terms from the Human Phenotype Ontology (HPO)

For each PubMed article which contains the word BASP1 in the title and/or abstract, we used the NCBO Annotator Web-service^59^ to identify mentions of disease terms from the HPO^58^ in the text as described in^69^. We configured the service to find the whole-word, direct terms matches for term labels and synonyms from the HPO. The HPO is indicated by the BioPortal version 5.3.1. The obtained word lists were clarified from non-informative terms: abnormality, disease, disorder, system, morphology, physiology, function and involving. Term lists were submitted to the online cloud generating software Wordle (http://www.wordle.net) and visualized using the “Horizontal” layout. Non-specific terms (such as Abnormality, Disease) are not used in tag-cloud representations.

## Electronic supplementary material

Supplementary information

Supplementary Datasets 1-28

## References

[1] Shoemaker BA, Panchenko AR (2007). Deciphering protein-protein interactions. Part II. Computational methods to predict protein and domain interaction partners. PLoS Comput. Biol..

[2] Kotlyar, M., Rossos, A. E. M. & Jurisica, I. In *Current Protocols in Bioinformatics* 8.2.1–8.2.14 (John Wiley & Sons, Inc., 10.1002/cpbi.38 (2017). 10.1002/cpbi.38 29220074

[3] Gemovic, B., Sumonja, J., Davidovic, R., Perovic, V., & Veljkovic, N. Mapping of Protein-Protein Interactions: Web-Based Resources for Revealing Interactomes. *Current Med Chem*, 10.2174/0929867325666180214113704 (2018). 10.2174/0929867325666180214113704 29446725

[4] Park Y, Marcotte EM (2012). Flaws in evaluation schemes for pair-input computational predictions. Nat. Methods.

[5] Hamp T, Rost B (2015). More challenges for machine-learning protein interactions. Bioinformatics.

[6] Wright PE, Dyson HJ (1999). Intrinsically unstructured proteins: re-assessing the protein structure-function paradigm. J. Mol. Biol..

[7] Uversky VN, Gillespie JR, Fink AL (2000). Why are ‘natively unfolded’ proteins unstructured under physiologic conditions?. Proteins Struct. Funct. Genet..

[8] Dunker AK (2001). Intrinsically disordered protein. J. Mol. Graph. Model..

[9] Williams RM (2001). The protein non-folding problem: amino acid determinants of intrinsic order and disorder. Pac. Symp. Biocomput..

[10] Uversky VN (2013). Intrinsic Disorder-based Protein Interactions and their Modulators. Curr. Pharm. Des..

[11] Dyson HJ, Wright PE (2002). Coupling of folding and binding for unstructured proteins. Curr. Opin. Struct. Biol..

[12] Wright PE, Dyson HJ (2015). Intrinsically disordered proteins in cellular signalling and regulation. Nat. Rev. Mol. Cell Biol..

[13] Tompa P, Fuxreiter M (2008). Fuzzy complexes: polymorphism and structural disorder in protein-protein interactions. Trends Biochem. Sci..

[14] Dyson HJ, Wright PE (2005). Intrinsically unstructured proteins and their functions. Nat. Rev. Mol. Cell Biol..

[15] Cuchillo R, Michel J (2012). Mechanisms of small-molecule binding to intrinsically disordered proteins. Biochem. Soc. Trans..

[16] Dunker AK, Brown CJ, Obradovic Z (2002). Identification and functions of usefully disordered proteins. Adv. Protein Chem..

[17] Guharoy M, Szabo B, Martos SC, Kosol S, Tompa P (2013). Intrinsic Structural Disorder in Cytoskeletal Proteins. Cytoskeleton.

[18] Uversky VN, Oldfield CJ, Dunker AK (2008). Intrinsically Disordered Proteins in HumanDiseases: Introducing the D ^2^ Concept. Annu. Rev. Biophys..

[19] Babu MM, van der Lee R, de Groot NS, Gsponer J (2011). Intrinsically disordered proteins: Regulation and disease. Curr. Opin. Struct. Biol..

[20] Krishnan N (2014). Targeting the disordered C terminus of PTP1B with an allosteric inhibitor. Nat. Chem. Biol..

[21] Hammoudeh DI, Follis AV, Prochownik EV, Metallo SJ (2009). Multiple independent binding sites for small-molecule inhibitors on the oncoprotein c-Myc. J. Am. Chem. Soc..

[22] Wass MN, Fuentes G, Pons C, Pazos F, Valencia A (2011). Towards the prediction of protein interaction partners using physical docking. Mol. Syst. Biol..

[23] Wodak SJ, Janin J (2017). Modeling protein assemblies: Critical Assessment of Predicted Interactions (CAPRI) 15 years hence. Proteins Struct. Funct. Bioinforma..

[24] Piovesan D (2017). DisProt 7.0: A major update of the database of disordered proteins. Nucleic Acids Res..

[25] Schaefer MH (2012). Hippie: Integrating protein interaction networks with experiment based quality scores. PLoS One.

[26] Haynes C (2006). Intrinsic disorder is a common feature of hub proteins from four eukaryotic interactomes. PLoS Comput. Biol..

[27] Patil A, Nakamura H (2006). Disordered domains and high surface charge confer hubs with the ability to interact with multiple proteins in interaction networks. FEBS Lett..

[28] Hu G, Wu Z, Uversky VN, Kurgan L (2017). Functional analysis of human hub proteins and their interactors involved in the intrinsic disorder-enriched interactions. Int. J. Mol. Sci..

[29] Ben-Hur A, Noble WS (2006). Choosing negative examples for the prediction of protein-protein interactions. BMC Bioinformatics.

[30] Park Y, Marcotte EM (2011). Revisiting the negative example sampling problem for predicting protein-protein interactions. Bioinformatics.

[31] Li W, Godzik A (2006). Cd-hit: A fast program for clustering and comparing large sets of protein or nucleotide sequences. Bioinformatics.

[32] Uversky VN, Dunker AK (2010). Understanding protein non-folding. Biochim. Biophys. Acta - Proteins Proteomics.

[33] Dosztányi Z, Csizmók V, Tompa P, Simon I (2005). The pairwise energy content estimated from amino acid composition discriminates between folded and intrinsically unstructured proteins. J. Mol. Biol..

[34] Mészáros B, Tompa P, Simon I, Dosztányi Z (2007). Molecular Principles of the Interactions of Disordered Proteins. J. Mol. Biol..

[35] Mao AH, Lyle N, Pappu RV (2013). Describing sequence–ensemble relationships for intrinsically disordered proteins. Biochem. J..

[36] Campen A (2008). TOP-IDP-Scale: A New Amino Acid Scale Measuring Propensity for Intrinsic Disorder. Protein Pept. Lett..

[37] Vihinen M, Torkkila E, Riikonen P (1994). Accuracy of protein flexibility predictions. Proteins Struct. Funct. Bioinforma..

[38] Galzitskaya OV, Garbuzynskiy SO, Lobanov MY (2006). FoldUnfold: Web server for the prediction of disordered regions in protein chain. Bioinformatics.

[39] Klein P, Kanehisa M, DeLisi C (1984). Prediction of protein function from sequence properties. Discriminant analysis of a data base. Biochim. Biophys. Acta.

[40] Chou KC (2001). Prediction of protein cellular attributes using pseudo-amino acid composition. Proteins.

[41] Martin S, Roe D, Faulon JL (2005). Predicting protein-protein interactions using signature products. Bioinformatics.

[42] Guo Y, Yu L, Wen Z, Li M (2008). Using support vector machine combined with auto covariance to predict protein-protein interactions from protein sequences. Nucleic Acids Res..

[43] Shen J (2007). Predicting protein-protein interactions based only on sequences information. Proc. Natl. Acad. Sci. USA.

[44] Gaudet P (2017). The neXtProt knowledgebase on human proteins: 2017 update. Nucleic Acids Res..

[45] Forsova OS, Zakharov VV (2016). High-order oligomers of intrinsically disordered brain proteins BASP1 and GAP-43 preserve the structural disorder. FEBS J..

[46] Toska E, Roberts SGE (2014). Mechanisms of transcriptional regulation by WT1 (Wilms’ tumour 1). Biochem. J..

[47] Yeoh E-J (2002). Classification, subtype discovery, and prediction of outcome in pediatric acute lymphoblastic leukemia by gene expression profiling. Cancer Cell.

[48] Moribe T (2008). Identification of novel aberrant methylation of BASP1 and SRD5A2 for early diagnosis of hepatocellular carcinoma by genome-wide search. Int. J. Oncol..

[49] Guo R-S (2016). Restoration of Brain Acid Soluble Protein 1 Inhibits Proliferation and Migration of Thyroid Cancer Cells. Chin. Med. J. (Engl)..

[50] Toska E, Shandilya J, Goodfellow SJ, Medler KF, Roberts SGE (2014). Prohibitin is required for transcriptional repression by the WT1–BASP1 complex. Oncogene.

[51] Marsh LA (2017). BASP1 interacts with oestrogen receptor α and modifies the tamoxifen response. Cell Death Dis..

[52] Hartl M, Nist A, Khan MI, Valovka T, Bister K (2009). Inhibition of Myc-induced cell transformation by brain acid-soluble protein 1 (BASP1). Proc. Natl. Acad. Sci..

[53] Carpenter B (2004). BASP1 is a transcriptional cosuppressor for the Wilms’ tumor suppressor protein WT1. Mol. Cell. Biol..

[54] Han M-H (2013). The Novel Caspase-3 Substrate Gap43 is Involved in AMPA Receptor Endocytosis and Long-Term Depression. Mol. Cell. Proteomics.

[55] Maere S, Heymans K, Kuiper M (2005). BiNGO: A Cytoscape plugin to assess overrepresentation of Gene Ontology categories in Biological Networks. Bioinformatics.

[56] Supek, F., Bošnjak, M., Škunca, N. & Šmuc, T. Revigo summarizes and visualizes long lists of gene ontology terms. *PLoS One* **6** (2011). 10.1371/journal.pone.0021800 PMC3138752 21789182

[57] Uversky VN (2011). Intrinsically disordered proteins from A to Z. Int. J. Biochem. Cell Biol..

[58] Köhler S (2017). The human phenotype ontology in 2017. Nucleic Acids Res..

[59] Whetzel PL (2011). BioPortal: Enhanced functionality via new Web services from the National Center for Biomedical Ontology to access and use ontologies in software applications. Nucleic Acids Res..

[60] Ransohoff KJ (2017). Two-stage genome-wide association study identifies a novel susceptibility locus associated with melanoma. Oncotarget.

[61] Kaehler KC (2014). Novel DNA methylation markers with potential prognostic relevance in advanced malignant melanoma identified using COBRA assays. Melanoma Res..

[62] Chinnery P (2000). Mitochondrial Disorders Overview. NCBI Bookshelf. A Serv. Natl. Libr. Med. Natl. Institutes Heal. Pagon.

[63] Geurts P, Ernst D, Wehenkel L (2006). Extremely randomized trees. Mach. Learn..

[64] Friedman JH (2001). Greedy function approximation: A gradient boosting machine. Ann. Stat..

[65] Friedman, J., Hastie, T. & Tibshirani, R. Regularization Paths for Generalized Linear Models via Coordinate Descent. *J. Stat. Softw*. **33**, (2010). PMC2929880 20808728

[66] Chang C, Lin C (2013). LIBSVM: A Library for Support Vector Machines. ACM Trans. Intell. Syst. Technol..

[67] RStudio Team, -. RStudio: Integrated Development for R. [Online] *RStudio, Inc., Boston, MA*, http//www.rstudio.com RStudio, Inc., Boston, MA 10.1007/978-81-322-2340-5 (2016).

[68] Bergstra J, Bengio Y (2012). Random Search for Hyper-Parameter Optimization. J. Mach. Learn. Res..

[69] LePendu P, Musen MA, Shah NH (2011). Enabling enrichment analysis with the Human Disease Ontology. J. Biomed. Inform..

