# Supplementary material for: IDPpi: Protein-Protein Interaction Analyses of Human Intrinsically Disordered Proteins
Source: Sci Rep. 2018 Jul 12;8:10563. doi: 10.1038/s41598-018-28815-x (PMC6043496; doi:10.1038/s41598-018-28815-x)
Supplement: Supplementary file 1 — Supplementary information [file 41598_2018_28815_MOESM1_ESM.docx]

*Supplementary*

**IDPpi: Protein-Protein Interaction Analyses of Human Intrinsically Disordered Proteins**

Vladimir Perovic^1^, Neven Sumonja^1^, Lindsey A. Marsh^2^, Sandro Radovanovic^3^, Milan Vukicevic^3^, Stefan G.E. Roberts^2^, Nevena Veljkovic^1^

^1^Centre for Multidisciplinary Research and Engineering, Vinca Institute of Nuclear Sciences, University of Belgrade, Belgrade, Serbia

^2^School of Cellular and Molecular Medicine, University of Bristol, Bristol, UK

^3^Centre for business decision making, Faculty of organizational Sciences, University of Belgrade, Belgrade, Serbia

**Supplementary Tables Descriptions**

Table S1. General PPI Training set 1 as provided in ^4^

Table S2. General PPI Training set 2 as provided in ^4^

Table S3. General PPI Training set 3 as provided in ^4^

Table S4. General PPI Training set 4 as provided in ^4^

Table S5. General PPI Training set 5 as provided in ^4^

Table S6. Performance comparison of different machine learning algorithms on the training datasets S1-S5

Table S7. IDP PPI Training set 1

Table S8. IDP PPI Training set 2

Table S9. IDP PPI Training set 3

Table S10. IDP PPI Training set 4

Table S11. IDP PPI Training set 5

Table S12. Test set 1 which corresponds to IDP PPI Training set 1

Table S13. Test set 2 which corresponds to IDP PPI Training set 2

Table S14. Test set 3 which corresponds to IDP PPI Training set 3

Table S15. Test set 4 which corresponds to IDP PPI Training set 4

Table S16. Test set 5 which corresponds to IDP PPI Training set 5

Table S17. Test set 1 which corresponds to IDP General Training set 1

Table S18. Test set 2 which corresponds to IDP General Training set 2

Table S19. Test set 3 which corresponds to IDP General Training set 3

Table S20. Test set 4 which corresponds to IDP General Training set 4

Table S21. Test set 5 which corresponds to IDP General Training set 5

Table S22. Test set 10N used in evaluation using a negative subsets randomly chosen from the negative set, where N is the size of the positive set.

Table S23. Test set 100N used in evaluation using a negative subsets randomly chosen from the negative set, where N is the size of the positive set.

Table S24. Literature data about BASP1 PPI

Table S25. Enriched gene ontology term annotations in known BASP1 interactions were identified using BinGO

Table S26. Enriched gene ontology term annotations in predicted BASP1 interactions were identified using BinGO

Table S27. Over-represented HPO term annotations in predicted BASP1 interactions

Table S28. Over-represented HPO terms annotations in BASP1 publications

Table S29. Amino acids encoding scales: TOP-IDP scale, B-values, the FoldUnfold scale, the DisProt scale, and the net charge scale
